# Global scientific trends on exosome research during 2007–2016: a bibliometric analysis

**DOI:** 10.18632/oncotarget.17223

**Published:** 2017-04-19

**Authors:** Yiran Wang, Qijin Wang, Xianzhao Wei, Jie Shao, Jian Zhao, Zicheng Zhang, Ziqiang Chen, Yushu Bai, Ning Wang, Yajie Wang, Ming Li, Xiao Zhai

**Affiliations:** ^1^ Department of Orthopedics, Changhai Hospital, Second Military Medical University, Shanghai, China; ^2^ Department of Oncology, Changhai Hospital, Second Military Medical University, Shanghai, China; ^3^ Department of Endocrinology, Changhai Hospital, Second Military Medical University, Shanghai, China; ^4^ Graduate Management Unit, Changhai Hospital, Second Military Medical University, Shanghai, China

**Keywords:** bibliometric, citation, H-index, exosome, VOSviewer

## Abstract

**Background:**

Exosomes are small vesicles of endosomal origin, and they can be used for the diagnosis and the treatment. However, limited data were for the evaluation of the trend of exosome researches. This study aims to investigate the trend of exosome researches and compare the contribution of research from different regions, organizations and authors.

**Methods:**

Exosome related publications from 2007 to 2016 were retrieved from the Web of Science database. Excel, GraphPad Prism 5 and VOSviewer software were used to analyze the research trend.

**Results:**

A total of 1852 papers were identified and were cited 62967 times. The United States accounted for 38.8% of the articles, 42.0% of the citations, and the highest H-index (76). China ranked the second in the number of articles, but the sixth in citation frequency (4337) and the fourth in H-index (36). The journals, *PLoS ONE* and *J Biol Chem* had the highest number of publications. The author, Gabrielsson S., has published the most papers in this field (22). The keyword “ribonucleic acid” was mentioned the most at 746 times, and the words, “stem cell”, “drug resistance” and “monocyte cell factor” were the latest hotspots appeared around 2015.

**Conclusion:**

Literature growth related to exosome is expanding rapidly. The quality of the articles from China still requires improvement. Recent studies focus on the relationship with tumor, and “stem cell”, “drug resistance” and “michigan cancer foundation-7” may be the newest topics that should be closely followed in exosome research.

## INTRODUCTION

Exosomes are small vesicles 30nm to 120 nm in diameter [1], defined by Johnstone in 1987 [2]. These secreted vesicles are present in many and perhaps all biological fluids, including blood, urine and cultured medium of cell cultures. It has become a hot topic since Valadi manifested that exosomes contain mRNAs, microRNAs, DNA fragments and proteins that have biological activities in 2007 [3, 4]. In addition, evidence is accumulating that exosomes can be used for diagnosis of renal function, Parkinson's disease and cancers like pancreatic cancer, breast cancer and colon cancer [5–10]. Meanwhile, there is a growing interest in the clinical applications of exosomes. And it is predicted that exosomes can be used for prognosis, for therapy, and as biomarkers for health and disease [11, 12]. Researches on exosomes have developed dramatically in the near years, but the evolution of scientific output in this field has been poorly explored to date and there are few internationally published reports on research activity in exosome.

Bibliometrics is defined as the application of statistics and mathematics for the analysis of written publications such as books and journal articles [13]. Bibliometrics is a good choice to evaluate the trend in research activity over time. Bibliometrics uses the literature system and literature metrology characteristics as research objects and analyzes the literatures quantitatively and qualitatively [14]. This type of analysis obtains the bibliographical works within a given field, topic, institution or country. It can provide an access to characterize the development in a certain field [15] and has helped a lot in govern policy making, clinical guideline and research trend in diabetes [16], respiratory medicine [17] and gastrointestinal diseases [18].

The aims of this study were to examine the publication pattern of exosome research output at the global level using the web of science (Thomson Reuters Company) since 2007. The geographic and temporal distribution of publications related to exosome were measured, together with the most used keywords, and the journals most commonly chosen. It is the latter application that we will highlight in this article, by using bibliometric mapping tools to map developments within exosome. Results were analyzed to better understand the field's structure or determining developments in exosome research.

## RESULTS

### Countries contributing to global publications

**Figure 1:**
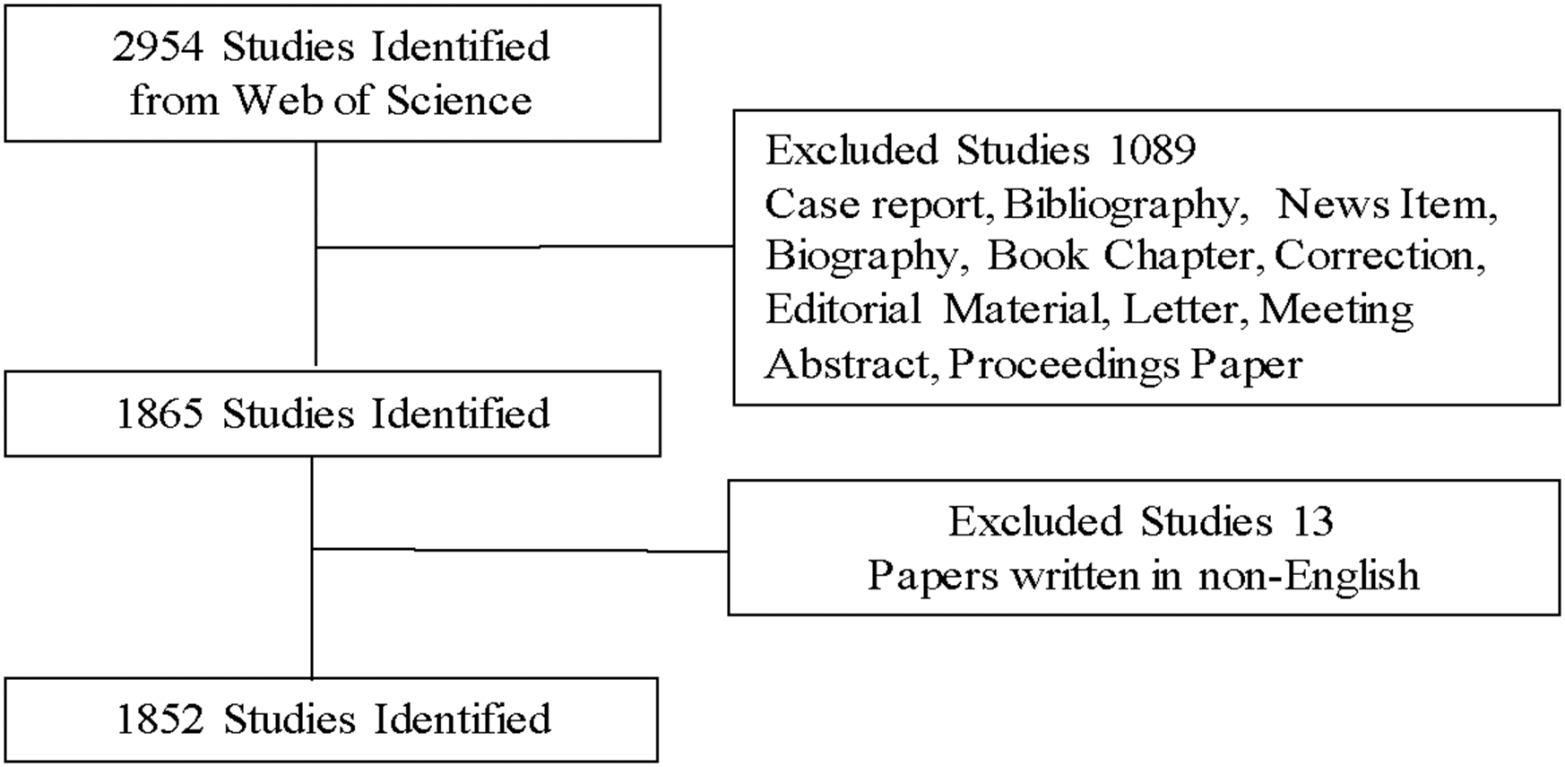
Flow diagram of exosome researches inclusion

**Figure 2:**
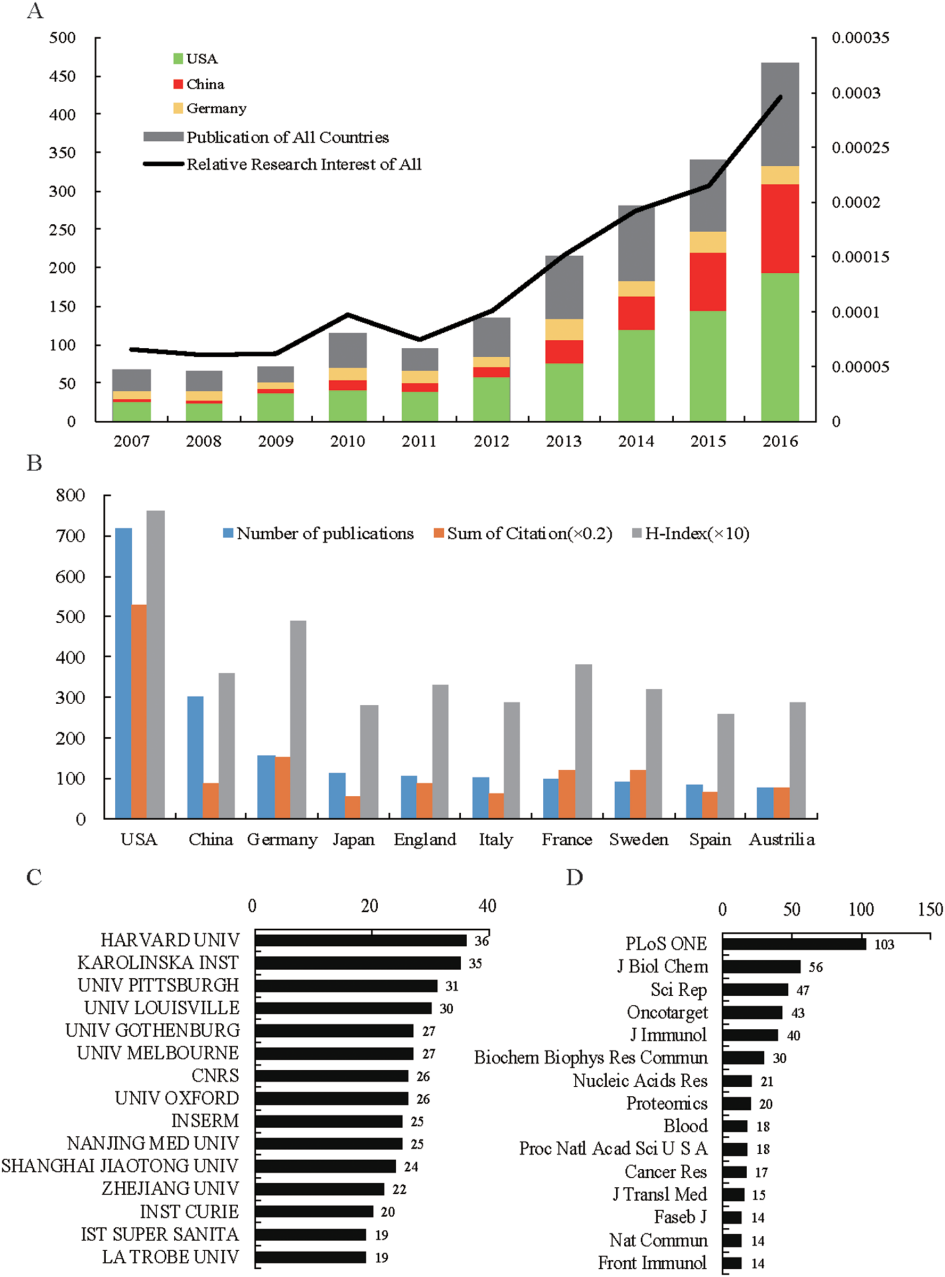
Contributive characteristics on exosome researches **(A)** The number of worldwide and the top 3 countries publications on exosome researches and the line of relative research interest; **(B)** The distribution, citation frequency (×0.2) and H-index data (×10) of publications in the top 10 countries; **(C)** The number of publications on exosome researches from the top 15 contribution institutions; **(D)** The number of publications in the top 15 popular journals on exosome researches.

A total of 1852 articles met the search criteria from 2007 to 2016 (Figure 1 and Supplementary Table 1). The relative research interest of exosome has grown year by year (Figure 2A). The United States published the most papers (719, 38.8%), followed by China (301, 16.3%) and Germany (157, 8.5%). The United States published the most papers per year (Figure 2A).

### Citation and H-index analysis

According to the analysis of the Web of Science database, all articles related to exosome had been cited 62967 times since 2007 (41379 times without self-citations). The cited frequency per paper was 33.8 times. The number of citations of papers from the United States was 26440, accounting for 42.0% of the total citations. The H-index of papers from the United States was 76. Germany ranked second with the citation frequency of 7670 and the H-index of 49. The number of publications of China ranked second, but the citation frequency and H-index ranked the sixth and forth respectively (Figure 2B).

### Distribution of institutes paying attention to exosomes

The institute with the greatest number of publications was *Harvard University* with a total of 36 papers, accounting for 1.94% of all published literature relating to the field. There were four French institutes in the top 15 institutes list. Meanwhile, 3 Chinese institutes, 3 American institutes, 2 Australian institutes, 1 English institute, 1 German institute and 1 Swedish institute were on the list (Figure 2C). Publications from top 15 institutes accounted for 21.24% of all literature on exosome.

### Distribution of published journals on exosomes

A quarter of the publications were published in the top 15 journals (470, 25.2%). The journal *PLoS ONE* (IF=3.057, 2016) published the most with 103 papers. Forty-two articles were published in the *Nature* and its series on exosome. The top 15 journals that published the most papers are shown in Figure 2D.

### Growth trends prediction of exosomes

**Figure 3:**
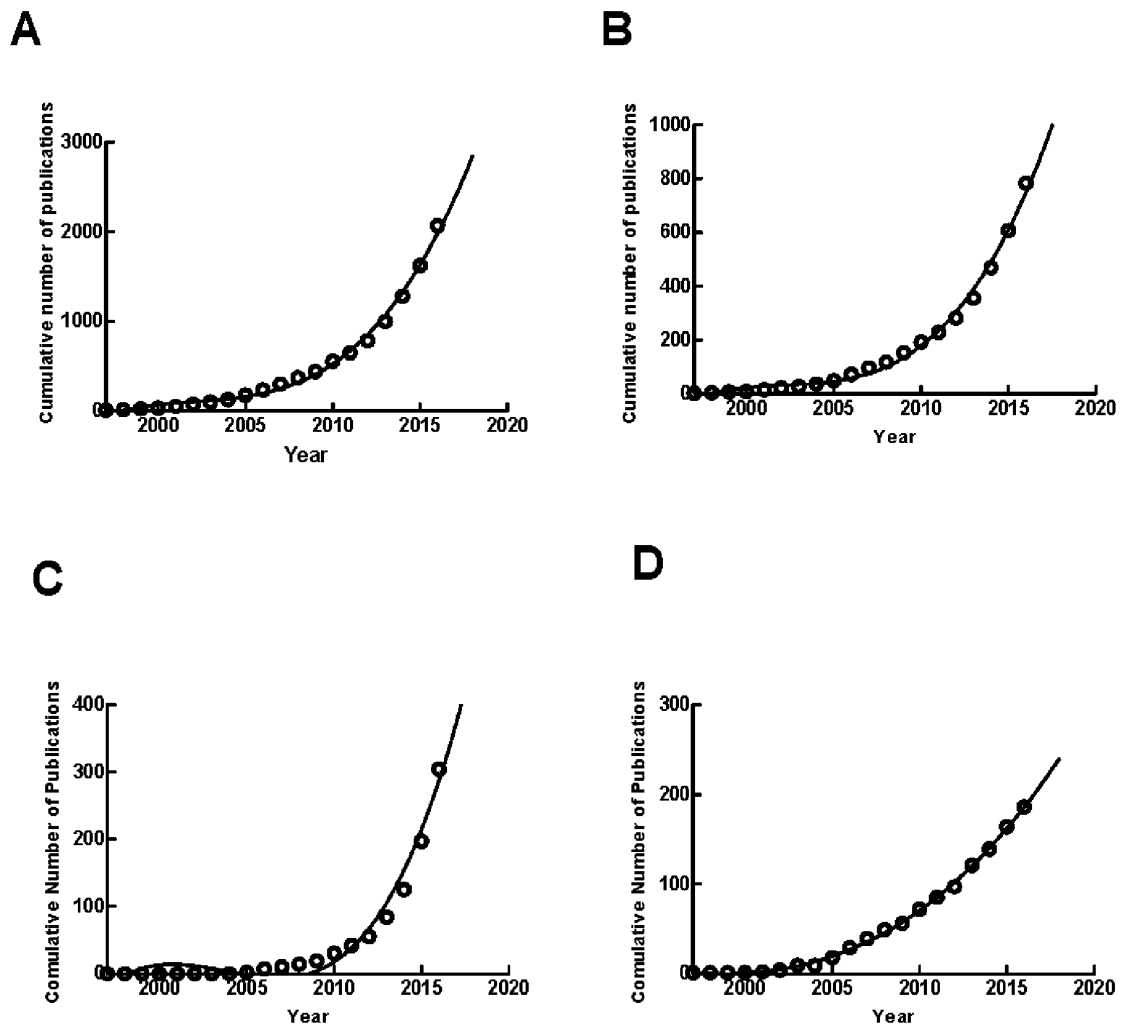
The model fitting curves of growth trends of exosome publications **(A)** Global; **(B)** the United States; **(C)** China; **(D)** Germany.

Model fitting curves of exosome publication growth showed a significant correlation between the year and cumulative number of exosome publications as in Figure 3. The publication number for 2017 was estimated by using cumulative publication numbers from 2007 to 2016. The worldwide, the United States, China and Germany were estimated to reach 2385, 908, 385 and 212 articles in 2017 respectively (Figure 3).

### Distribution of authors on exosomes

Top 10 authors contributed a total of 164 papers relating to exosome, accounting for 8.8% of all published literature relating to the field. Gabrielsson S. from Karolinska Institute in Sweden published the most papers in this field (22 papers), followed by Zhang H.G. from University of Louisville in the United States with 20 publications and Conti E. with 18 publications. Xu WR from Jiangsu University and Wang Y from Shanghai Jiaotong Univeristy were the most productive authors in China (Table 1).

**Table 1:** Top 10 authors with the most publications related to exosome research

Author	Number of Papers	Country	Affiliations
Gabrielsson S	22	Sweden	Department of Medicine Solna, Clinical Allergy Research Unit, Karolinska Institutet
Zhang HG	20	USA	Univ Louisville, Brown Canc Ctr, Dept Microbiol & Immunol, Louisville
Conti E	18	Germany	Department of Structural Cell Biology, Max Planck Institute of Biochemistry, Martinsried
Xu WR	17	China	School of Medicine, Jiangsu University
Wang Y	17	China	Institute of Microsurgery on Extremities, Shanghai Jiao Tong University Affiliated Sixth People's Hospital
Simpson RJ	16	Australia	La Trobe Univ, La Trobe Inst Mol Sci, Dept Biochem, Bundoora
Qian Hui	16	China	School of Medicine, Jiangsu University
Thery C	15	France	Inst Curie, Ctr Rech, Paris
Zhang X	15	China	School of Medicine, Jiangsu University
Whiteside TL	14	USA	Univ Pittsburgh, Inst Canc, Pittsburgh

### Hotspots of studies on exosomes

**Figure 4:**
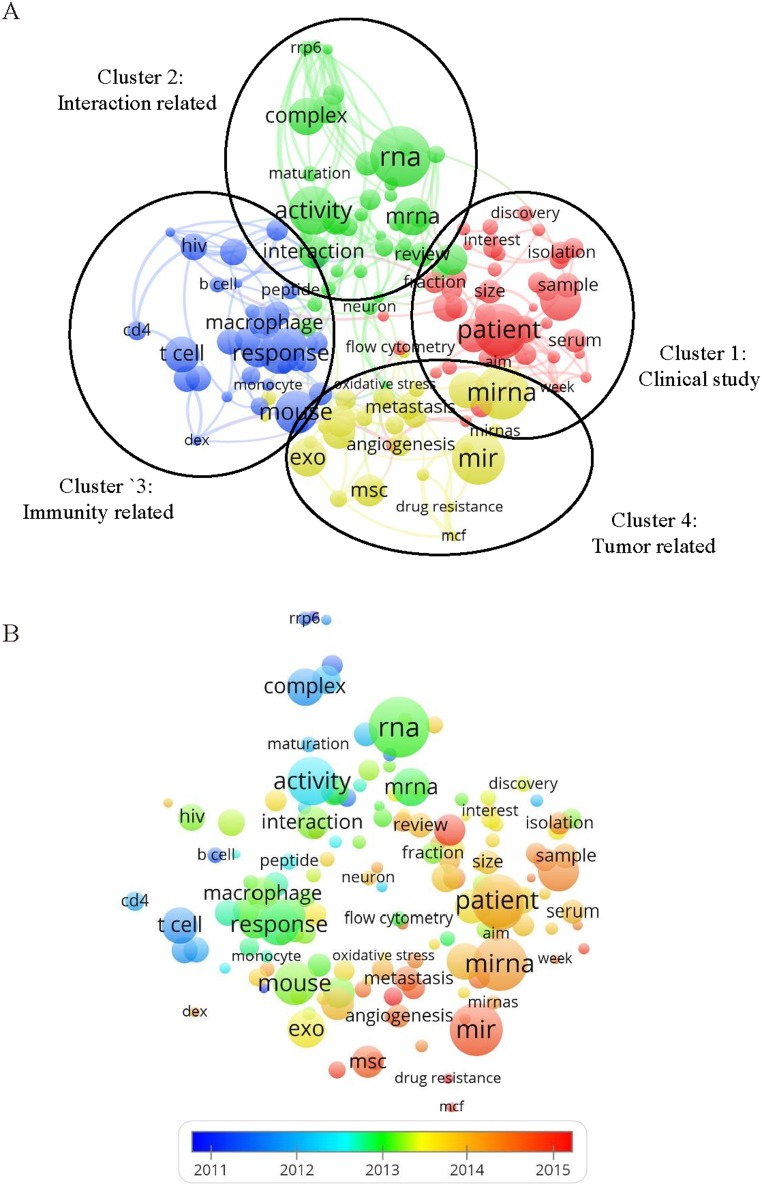
The analysis of key words **(A)** Mapping on key words of exosome, the keywords were divided into four clusters according to different colors generated by default: Clinical Study (right in red), interaction related study (up in green), immunity related study (left in blue) and tumor related study (down in yellow). A large size of a circle represents the keyword appears at a high frequency; **(B)** Distribution of key words according to when they appeared for the average time, key words in blue presented earlier than those in yellow or red. Two terms are defined to co-occur if they both occur on the same line in the corpus file. In general, the smaller the distance between two terms, the larger the number of co-occurrences of the terms.

Keywords used in the 1852 papers were analyzed through VOSviewer. As shown in Figure 4A, the 150 keywords (defined as being used more than 45 times within titles and abstracts in all of articles) were classified to four clusters: “clinical study”, “interaction related research”, “immunity related research” and “tumor related research”. Among the “interaction related research” cluster, keywords used in the publications of exosome were listed as follows: RNA (746 times), mRNA (384 times), complex (368 times). For the immunity related research, the primary keywords were as follows: mouse (1489 times), response (437 times), T cell (348 times). For the clinical study, the main keywords were as follows: patient (607 times), biomarker (426 times), plasma (243 times). For the tumor related research, the keywords were as follows: miRNA (643 times), proliferation (314 times), and metastasis (209 times) (Supplementary Table 2).

In Figure 4B, VOSViewer applied colors to key words based upon when they appeared in literature for the average time. Key words in blue color appeared early, and key words in red color appeared recently. In early stage of exosome research, immune response (cluster 3, the average appearing year (AAY) of keywords is 2013.0) and intracellular interaction (cluster 2, AAY is 2012.9) were the main hotspots. Recent trend showed that in tumor related research (cluster 4, AAY is 2014.1), “stem cell” and “drug resistance” appeared in 2015 and 2014 as keywords for 135 and 63 times respectively, and in clinical study (cluster 1, AAY is 2013.7), “serum exosome” was the recent keyword for 67 times and it was appeared in 2014.

## DISCUSSION

### Global trends of researches on exosomes

The United States and Germany ranked the first and the second for citation frequency in exosome research, respectively. Additionally, the number of the publications and H-index of the United States were higher than that of any other countries, suggesting that American and German scientists have published many high quality articles, and have taken the leading position regarding exosome research. In addition, China ranked second in total number of articles for many years, but sixth in citation frequency and forth in H-index. England, France and Sweden had fewer publications than that of China, but their citation frequency and H-index were higher than that of China. This suggested that more efforts should be taken to raise the influence of Chinese research.

Additionally, there was a rapid growth of publications related to exosome research from 2007 to 2016 all over the world. The time curves showed that there might be more publications in this field in next years. The number of publications in recent years rapidly surpasses the total number of publications in the past.

French institutes were the leading organizations on exosome research in the quantity. There were four French institutes on the top 15 institutes list. However, the most productive institution was *Harvard University*. Meanwhile, three American were on the list, as well as three Chinese institutes. The reason why the United States had the most publications in this field was that it had the most powerful institutions around the world.

It is of note that the Journals *PLoS ONE* has been far ahead with 103 articles, additionally *Journal of Biological Chemistry, Scientific Reports* and *Oncotarget* were the main journals involved in publishing exosome papers. Moreover, it indicated that future development within exosome would likely be released within the aforementioned journals.

In terms of authors and publications, Gabrielsson S. from Sweden, Zhang H.G. from the United States and Conti E. form Germany had published the most articles on exosome. Gabrielsson S. looked into the immune response and cancer immunotherapy of exosome [19, 20]. Zhang H.G. focused on the communications of cells by exosome [21, 22]. The articles of Conti E. were mainly related to the effect of RNA in exosome [23, 24]. These scientists were leaders in the exosome research, and their studies may still have a huge impact on exosome research in future and help design our own experiments. They are excellent candidates for cooperation.

### Research focuses on exosomes

Articles that were cited the most on exosome were of regarded as the fundamental basis for further studies. The details of top 10 cited studies were shown in Table 2. In the cluster of “interaction related researches”, “RNA” (746 times, averagely appeared in 2013) and “mRNA” (384 times, averagely appeared in 2014) were the most used. The article titled “Exosome-mediated transfer of mRNAs and microRNAs is a novel mechanism of genetic exchange between cells” was the most cited for 665 times. It was published in Nature Cell Biology in 2007 [3]. The article found that exosomes contain both mRNA and microRNA, which can be delivered to another cell, and can be functional in this new location. For the latest hotspots, “stem cell” (2015.0), “drug resistance” (2015.0) and “MCF” (2014.9, michigan cancer foundation-7 in human breast adenocarcinoma cell line) were averagely appeared the most recently.

**Table 2:** Top 10 studies with the most citation frequency related to exosome research

Title	First author	Journal	Year	Citation frequency per year	Main Conclusion
Exosome-mediated transfer of mRNAs and microRNAs is a novel mechanism of genetic exchange between cells	Valadi, Hadi	NATURE CELL BIOLOGY	2007	255.73	Exosomes contain both mRNA and microRNA, which can be delivered to another cell, and can be functional in this new location.
Extracellular vesicles: Exosomes, microvesicles, and friends	Raposo, Graca	JOURNAL OF CELL BIOLOGY	2013	147.8	In this review, they focus on the characterization of EVs and on currently proposed mechanisms for their formation, targeting, and function.
Melanoma exosomes educate bone marrow progenitor cells toward a pro-metastatic phenotype through MET	Peinado, Hector	NATURE MEDICINE	2012	119.67	Exosome production, transfer and education of bone marrow cells supports tumor growth and metastasis
Delivery of siRNA to the mouse brain by systemic injection of targeted exosomes	Alvarez-Erviti, Lydia	NATURE BIOTECHNOLOGY	2011	98.43	The therapeutic potential of exosome-mediated siRNA delivery was demonstrated by the strong mRNA (60%) and protein (62%) knockdown of BACE1, a therapeutic target in Alzheimer's disease, in wild-type mice.
MicroRNA signatures of tumor-derived exosomes as diagnostic biomarkers of ovarian cancer	Taylor, Douglas D.	GYNECOLOGIC ONCOLOGY	2008	82	icroRNA profiling of circulating tumor exosomes could potentially be used as surrogate diagnostic markers
Ceramide triggers budding of exosome vesicles into multivesicular Endosomes	Trajkovic, Katarina	SCIENCE	2008	70.4	Purified exosomes were enriched in ceramide, and the release of exosomes was reduced after the inhibition of neutral sphingomyelinases.
Exosomes: Extracellular organelles important in intercellular communication	Mathivanan, Suresh	JOURNAL OF PROTEOMICS	2010	65.12	In this review they focus on various strategies for purifying exosomes and discuss their biophysical and biochemical properties.
Functional delivery of viral miRNAs via exosomes	Pegtel, D. Michiel	PROCEEDINGS OF THE NATIONAL ACADEMY OF SCIENCES OF THE UNITED STATES OF AMERICA	2010	65	miRNA-mediated gene silencing as a potential mechanism of intercellular communication between cells of the immune system may be exploited by the persistent human gamma-herpesvirus EBV
Unidirectional transfer of microRNA-loaded exosomes from T cells to antigen-presenting cells	Mittelbrunn, Maria;	NATURE COMMUNICATIONS	2011	64.29	Cellular communication involving antigen-dependent, unidirectional intercellular transfer of miRNAs may be related to exosomes during immune synapsis
Biogenesis, Secretion, and Intercellular Interactions of Exosomes and Other Extracellular Vesicles	Colombo, Marina;	ANNUAL REVIEW OF CELL AND DEVELOPMENTAL BIOLOGY, VOL 30	2014	63.75	This review focuses on the definition of exosomes and other secreted extracellular vesicles.

In Figure 4 A and B, we can find that the emphasis of exosome research has shifted mainly from basic studies to clinical studies in the recent years. It is in accordance with the law of the development of a new discipline and translational medicine. As the rudimentary knowledge of the exosome was recognized, its application has then been followed for illumination. For clinical studies, the exosome was used mainly for diagnosis as a biomarker from the serum of patients. We believed that exosome might be also a treatment agent in the future. As a result, the development of researches on exosomes is very fast, and the translational research might be the next breakthrough point.

### Strengths and limitations

Papers on exosome evaluated in this study were reviewed from the Web of Science (WoS) database of Science Citation Index Expanded journals. The data analysis was relatively comprehensive and objective. However, there are some limitations. Papers in non-English languages may be not included in the database and may have excluded important non-English research studies in exosome. Other databases were not analyzed such as Scopus, Google Scholar or PubMed, since WoS has gone a step further in presenting metadata lists, including author, source, year, document, countries and institutions types. Secondly, there were still differences between the real research conditions and bibliometric analysis results, since some recent published papers do not have high citation frequency. Thirdly, the global analysis was not adjusted for economic condition, population size, technology progress and other factors. Last, though all searches were conducted on January 10, 2017 to avoid bias due to the updating of the database for year 2016 since the database is still open for new data. However, we think the database had included the vast majority of articles published in 2016 and the additional small amount of new data might not change the conclusion.

## CONCLUSION

This study helps scientists master the trends of exosome research. The number of publications of exosomes will grow rapidly in the near future. The United States made the largest contribution in the exosome research. Though quantity of China ranked No.2, the quality and influence still require improvements. *PLoS ONE* and *J Biol Chem* were with the most relating publications. Gabrielsson S., Zhang H.G. and Conti E. may be good candidates for collaborative research in this field. All publications can be divided into 4 clusters. In the relatively new “tumor related” cluster, “stem cell”, “drug resistance” and “MCF” may be the latest hot spots, and related researches may be pioneers to lead this field in the next few years.

## MATERIALS AND METHODS

### Sources of the data and search strategy

The data in this article were based on the Science Citation Index-Expanded (SCI-E) of the Thomson Reuters Web of Science from 2007 to 2016. It is set after year 2007 since we aimed to investigate the recent global scientific trend after Valadi published his revolutionary results. A comprehensive online search was performed. The data were downloaded from the public databases and as secondary data, did not involve any interactions with human subjects. There were no ethical questions about the data. Ethical approval was not necessary.

All searches were conducted on a single day, January 10, 2017 to avoid bias due to the daily updating of the database as much as possible. The following search key words were used: TI= (exosom*) OR TI= (exosc*) NOT TI= (exoscreen) NOT TI= (exoscop*) NOT TI= (exosca*) AND Language = English. While there are a variety of manuscript types, only peer-reviewed articles and reviews are included.

### Data collection

The data were extracted from all eligible publications independently by two authors (Wang Yiran and Zhai Xiao). The txt data download from Web of Science were imported into Microsoft Excel 2016, GraphPad Prism 5 and VOSviewer. The data were analyzed both quantitatively and qualitatively.

Bibliometric indicators were extracted from the data, including publication number, citation frequency, relative research interest, H-index [25, 26], and so on. The relative research interest was designed as the quantity of weighted publications per year divided by the number of publications across all disciplines per year [27]. H-index is calculated as a measure of scientific research impact that reflects both the number of publications and the number of citations per publication: a scholar has published H papers, each of which has been cited in other papers at least H times [28].

### Statistical methods

GraphPad Prism 5 (GraphPad Prism Software Inc., San Diego, California) was used to analyze the time trend of the publications. The model: *f(x)* = *ax*^3^+*bx*^2^+*c*^x^+*d* was used to calculate the cumulative volume and to predict future trend of papers in this field. Symbol x represents the year, and *f(x)* was the cumulative volume of paper by the year.

VOSviewer (Leiden University, Leiden, Netherlands) was used to analyze the relations among highly cited references and productive authors. It is commonly used for mapping and clustering of co-citation network analysis. It also clusters citation terms and portrays the key words by color. The density of occurrence of information is portrayed by the size of the circle [29].

## SUPPLEMENTARY MATERIALS TABLES







## Abbreviations

MCF: Michigan Cancer Foundation - 7
