# Supplementary material for: Global scientific trends on exosome research during 2007–2016: a bibliometric analysis
Source: Oncotarget. 2017 Apr 19;8(29):48460–70. doi: 10.18632/oncotarget.17223 (PMC5564662; doi:10.18632/oncotarget.17223)
Supplement: Supplementary file 1 [file oncotarget-08-48460-s001.pdf]

Global scientific trends on exosome research during 2007–2016: a bibliometric analysis

SUPPLEMENTARY TABLES

Supplementary Table 1: Details of the total 1852 studies

See Supplementary File 1

Supplemental Table 2: Details of Group Items by Cluster in VOSviewer

See Supplementary File 2
