# Supplementary material for: Global scientific trends on exosome research during 2007–2016: a bibliometric analysis
Source: Oncotarget. 2017 Apr 19;8(29):48460–70. doi: 10.18632/oncotarget.17223 (PMC5564662; doi:10.18632/oncotarget.17223)
Supplement: Supplementary file 3 [file oncotarget-08-48460-s003.docx]

| \| Supplemental Table 2. Details of Group Items by Cluster in VOSviewer \| \| --- \| | | | | | | |
| --- | --- | --- | --- | --- | --- | --- | --- |
|  | label | Weight  <Occurrences> | weight<Co-occurrences> | cluster | score<Avg. pub. year> | score<Avg. cit. impact> |
| 1 | activation | 322 | 5023 | 3 | 2013.04 | 0.9186 |
| 2 | activity | 548 | 7058 | 2 | 2012.447 | 0.7762 |
| 3 | aim | 93 | 1608 | 1 | 2014.086 | 0.9751 |
| 4 | alpha | 139 | 1907 | 3 | 2013.144 | 1.2192 |
| 5 | angiogenesis | 188 | 2778 | 4 | 2014.372 | 1.2216 |
| 6 | antigen | 206 | 3240 | 3 | 2012.767 | 0.6358 |
| 7 | apoptosis | 188 | 3007 | 3 | 2013.08 | 0.7594 |
| 8 | application | 179 | 2327 | 1 | 2014.246 | 1.0464 |
| 9 | association | 89 | 1078 | 2 | 2011.775 | 1.0388 |
| 10 | b cell | 111 | 1703 | 3 | 2011.505 | 0.8022 |
| 11 | beta | 170 | 2157 | 3 | 2013.494 | 1.039 |
| 12 | biogenesis | 159 | 1732 | 2 | 2013.233 | 1.4073 |
| 13 | biomarker | 426 | 6930 | 1 | 2014.27 | 1.1585 |
| 14 | blood | 165 | 2926 | 1 | 2013.285 | 1.1664 |
| 15 | body fluid | 100 | 1605 | 1 | 2013.52 | 1.1798 |
| 16 | breast cancer | 69 | 1246 | 4 | 2014.58 | 1.0403 |
| 17 | breast cancer cell | 77 | 1248 | 4 | 2014.299 | 0.9601 |
| 18 | cancer patient | 99 | 1686 | 1 | 2012.929 | 1.2764 |
| 19 | cancer progression | 71 | 989 | 4 | 2014.465 | 1.0811 |
| 20 | cd4 | 149 | 3422 | 3 | 2012.054 | 0.7267 |
| 21 | cd81 | 65 | 1103 | 1 | 2012.308 | 0.9597 |
| 22 | characterization | 144 | 2052 | 1 | 2013.465 | 0.9591 |
| 23 | combination | 57 | 838 | 1 | 2013.14 | 0.7541 |
| 24 | comparison | 57 | 1015 | 1 | 2013.509 | 0.6653 |
| 25 | complex | 368 | 4036 | 2 | 2011.902 | 0.672 |
| 26 | component | 245 | 3126 | 2 | 2012.988 | 0.9464 |
| 27 | composition | 185 | 2239 | 2 | 2013.389 | 1.0683 |
| 28 | concentration | 169 | 2830 | 1 | 2013.793 | 0.8766 |
| 29 | contrast | 84 | 1311 | 2 | 2012.333 | 0.8056 |
| 30 | control | 256 | 4114 | 1 | 2013.758 | 0.898 |
| 31 | cytokine | 130 | 2214 | 3 | 2013.692 | 0.8614 |
| 32 | day | 71 | 1266 | 1 | 2013.606 | 0.6847 |
| 33 | dcs | 226 | 3803 | 3 | 2011.912 | 0.8488 |
| 34 | degradation | 248 | 3028 | 2 | 2012.149 | 0.6934 |
| 35 | dendritic cell | 214 | 3669 | 3 | 2012.005 | 0.8254 |
| 36 | detection | 160 | 2448 | 1 | 2014.231 | 1.2634 |
| 37 | dex | 63 | 1184 | 3 | 2014.079 | 1.1266 |
| 38 | diagnosis | 186 | 2911 | 1 | 2014.376 | 1.2573 |
| 39 | difference | 92 | 1357 | 1 | 2013.63 | 1.1447 |
| 40 | differentiation | 161 | 2178 | 4 | 2013.609 | 0.9927 |
| 41 | discovery | 108 | 1469 | 1 | 2013.352 | 0.833 |
| 42 | drug resistance | 63 | 1005 | 4 | 2014.984 | 1.033 |
| 43 | ebv | 51 | 681 | 3 | 2012.647 | 1.2029 |
| 44 | endothelial cell | 173 | 2133 | 4 | 2014 | 1.1933 |
| 45 | enzyme | 82 | 1158 | 2 | 2012.61 | 0.9636 |
| 46 | exo | 381 | 6864 | 4 | 2013.465 | 0.7922 |
| 47 | exosomal mirna | 59 | 1185 | 1 | 2014.305 | 1.0171 |
| 48 | exosomal protein | 74 | 977 | 1 | 2013.662 | 0.8902 |
| 49 | exosome release | 104 | 1201 | 2 | 2013.308 | 0.9873 |
| 50 | exosome secretion | 103 | 1018 | 2 | 2013.689 | 0.8084 |
| 51 | extracellular space | 64 | 725 | 2 | 2013.031 | 1.1343 |
| 52 | extracellular vesicle | 295 | 3693 | 2 | 2014.78 | 1.4914 |
| 53 | flow cytometry | 99 | 1931 | 1 | 2012.99 | 1.0838 |
| 54 | fold | 53 | 870 | 1 | 2013.623 | 0.9555 |
| 55 | fraction | 174 | 2730 | 1 | 2013.195 | 1.0959 |
| 56 | fusion | 85 | 1075 | 2 | 2012.706 | 1.1627 |
| 57 | group | 168 | 2811 | 1 | 2014.423 | 0.8655 |
| 58 | hiv | 237 | 3441 | 3 | 2013.173 | 0.7508 |
| 59 | identification | 106 | 1526 | 1 | 2013.368 | 1.0218 |
| 60 | immune cell | 87 | 1373 | 3 | 2013.678 | 1.4345 |
| 61 | immune response | 215 | 3441 | 3 | 2012.763 | 0.6866 |
| 62 | induction | 135 | 2112 | 3 | 2012.83 | 0.8742 |
| 63 | infection | 229 | 3514 | 3 | 2013.266 | 0.6993 |
| 64 | inflammation | 113 | 1791 | 3 | 2013.566 | 1.0258 |
| 65 | inhibition | 132 | 1803 | 2 | 2013.159 | 0.9373 |
| 66 | interaction | 312 | 3831 | 2 | 2013.176 | 0.9103 |
| 67 | interest | 93 | 1381 | 1 | 2013.796 | 1.267 |
| 68 | invasion | 115 | 1628 | 4 | 2014.287 | 0.9208 |
| 69 | isolation | 178 | 3015 | 1 | 2014.202 | 1.195 |
| 70 | knowledge | 78 | 987 | 2 | 2013.487 | 1.2686 |
| 71 | lipid | 143 | 1604 | 2 | 2014.091 | 1.2439 |
| 72 | loss | 89 | 1134 | 2 | 2013.169 | 0.9062 |
| 73 | macrophage | 300 | 4195 | 3 | 2013.15 | 0.7297 |
| 74 | marker | 319 | 5262 | 1 | 2013.549 | 0.9395 |
| 75 | maturation | 105 | 1264 | 2 | 2012.143 | 0.6115 |
| 76 | mcf | 54 | 798 | 4 | 2014.852 | 0.8434 |
| 77 | mesenchymal stem cell | 140 | 2116 | 4 | 2014.743 | 1.2399 |
| 78 | metastasis | 209 | 2490 | 4 | 2014.459 | 1.5613 |
| 79 | method | 535 | 8764 | 1 | 2013.974 | 1.0518 |
| 80 | microrna | 360 | 5905 | 4 | 2013.892 | 1.5018 |
| 81 | migration | 187 | 2516 | 4 | 2014.583 | 0.8829 |
| 82 | mir | 615 | 10099 | 4 | 2014.646 | 1.2593 |
| 83 | mirna | 643 | 9900 | 4 | 2014.123 | 1.5305 |
| 84 | mirnas | 92 | 1642 | 4 | 2014.174 | 1.3805 |
| 85 | monocyte | 77 | 1285 | 3 | 2012.727 | 0.8459 |
| 86 | morphology | 65 | 1057 | 1 | 2013.092 | 0.6833 |
| 87 | mouse | 462 | 7523 | 3 | 2013.18 | 1.0161 |
| 88 | mrna | 384 | 5127 | 2 | 2012.945 | 1.0759 |
| 89 | msc | 305 | 4496 | 4 | 2014.551 | 1.5257 |
| 90 | mscs | 126 | 2072 | 4 | 2014.778 | 1.6927 |
| 91 | multivesicular body | 126 | 1651 | 2 | 2012.111 | 1.1576 |
| 92 | mutation | 126 | 1258 | 2 | 2013.714 | 0.9159 |
| 93 | mvb | 60 | 832 | 2 | 2012.75 | 0.8874 |
| 94 | nanoparticle tracking analysis | 48 | 792 | 1 | 2014.438 | 0.9107 |
| 95 | nef | 53 | 1105 | 3 | 2013.811 | 0.7613 |
| 96 | neuron | 103 | 1023 | 2 | 2013.854 | 1.2836 |
| 97 | nucleic acid | 106 | 1345 | 2 | 2014.028 | 1.1679 |
| 98 | oxidative stress | 52 | 657 | 2 | 2013.404 | 1.3093 |
| 99 | particle | 140 | 2001 | 2 | 2013.093 | 1.0306 |
| 100 | pathogenesis | 109 | 1410 | 3 | 2014.028 | 0.954 |
| 101 | patient | 607 | 10053 | 1 | 2013.908 | 1.2541 |
| 102 | peptide | 117 | 1674 | 3 | 2012.573 | 0.9938 |
| 103 | plasma | 243 | 4442 | 1 | 2013.7 | 1.0121 |
| 104 | plasma membrane | 136 | 1599 | 2 | 2011.706 | 1.0356 |
| 105 | pregnancy | 81 | 1254 | 1 | 2013.79 | 0.8521 |
| 106 | processing | 164 | 1898 | 2 | 2011.47 | 0.6726 |
| 107 | production | 214 | 3095 | 3 | 2013.065 | 0.9867 |
| 108 | proliferation | 314 | 4921 | 4 | 2013.866 | 0.8947 |
| 109 | prostate cancer | 71 | 1191 | 1 | 2013.747 | 1.3669 |
| 110 | proteomic analysis | 85 | 1112 | 1 | 2013.118 | 0.9964 |
| 111 | rat | 89 | 1659 | 1 | 2014.483 | 1.0861 |
| 112 | response | 437 | 6736 | 3 | 2012.892 | 0.8553 |
| 113 | review | 203 | 2199 | 2 | 2013.847 | 1.3616 |
| 114 | rna | 746 | 9515 | 2 | 2013.047 | 1.2403 |
| 115 | rnas | 97 | 1170 | 2 | 2013.258 | 0.8845 |
| 116 | rrp44 | 61 | 689 | 2 | 2011.885 | 0.7121 |
| 117 | rrp6 | 91 | 1147 | 2 | 2011.582 | 0.4761 |
| 118 | saliva | 81 | 1382 | 1 | 2012.161 | 1.2808 |
| 119 | sample | 206 | 3642 | 1 | 2014.078 | 1.1127 |
| 120 | serum | 189 | 3349 | 1 | 2013.884 | 1.1507 |
| 121 | serum exosome | 67 | 1279 | 1 | 2014.761 | 0.9222 |
| 122 | sirna | 72 | 829 | 2 | 2014.097 | 1.3054 |
| 123 | size | 196 | 2733 | 1 | 2013.781 | 0.9851 |
| 124 | source | 156 | 2648 | 1 | 2013.564 | 1.0626 |
| 125 | stem cell | 135 | 1772 | 4 | 2015.03 | 1.0906 |
| 126 | stimulation | 92 | 1449 | 3 | 2013.098 | 0.7089 |
| 127 | structure | 196 | 2128 | 2 | 2012.168 | 0.7003 |
| 128 | t cell | 348 | 6120 | 3 | 2011.842 | 0.7321 |
| 129 | tex | 104 | 1675 | 4 | 2014.067 | 0.7587 |
| 130 | tgf beta | 93 | 1567 | 4 | 2013.613 | 1.2152 |
| 131 | tnf alpha | 67 | 1249 | 3 | 2012.537 | 0.4829 |
| 132 | transmission electron microscopy | 62 | 1122 | 1 | 2014.032 | 0.8928 |
| 133 | tumor cell | 287 | 4406 | 4 | 2013.174 | 0.8265 |
| 134 | tumor exosome | 57 | 1009 | 4 | 2011.14 | 1.4563 |
| 135 | tumor growth | 89 | 1446 | 4 | 2013.258 | 1.2948 |
| 136 | tumor microenvironment | 113 | 1608 | 4 | 2014.416 | 1.1954 |
| 137 | tumor progression | 69 | 1057 | 4 | 2013.71 | 1.056 |
| 138 | tumour | 58 | 849 | 4 | 2013.569 | 1.7445 |
| 139 | ultracentrifugation | 141 | 2558 | 1 | 2013.255 | 0.8309 |
| 140 | urinary exosome | 151 | 2379 | 1 | 2013.185 | 0.8345 |
| 141 | urine | 150 | 2555 | 1 | 2013.453 | 1.0865 |
| 142 | use | 165 | 2194 | 1 | 2013.958 | 1.2229 |
| 143 | variety | 109 | 1413 | 2 | 2013.404 | 0.8419 |
| 144 | virus | 170 | 2191 | 3 | 2013.582 | 0.6859 |
| 145 | vitro | 327 | 5234 | 3 | 2013.04 | 0.9227 |
| 146 | vivo | 174 | 2564 | 3 | 2013.207 | 1.2337 |
| 147 | week | 50 | 904 | 1 | 2014.38 | 0.6318 |
| 148 | western blotting | 66 | 1215 | 1 | 2013.606 | 0.7176 |
| 149 | work | 78 | 964 | 2 | 2013.154 | 0.8965 |
| 150 | yeast | 64 | 730 | 2 | 2010.781 | 0.4742 |
